# Immunogenic and Invasive Properties of *Brucella melitensis* 16M Outer Membrane Protein Vaccine Candidates Identified via a Reverse Vaccinology Approach

**DOI:** 10.1371/journal.pone.0059751

**Published:** 2013-03-22

**Authors:** Gabriel Gomez, Jianwu Pei, Waithaka Mwangi, L. Garry Adams, Allison Rice-Ficht, Thomas A. Ficht

**Affiliations:** 1 Department of Veterinary Pathobiology, Texas A&M University, College Station, Texas, United States of America; 2 Department of Cellular and Molecular Medicine, Texas A&M University Health Science Center, College Station, Texas, United States of America; Tulane University, United States of America

## Abstract

*Brucella* is the etiologic agent of brucellosis, one of the most common and widely distributed zoonotic diseases. Its highly infectious nature, the insidious, systemic, chronic, debilitating aspects of the disease and the lack of an approved vaccine for human use in the United States are features that make *Brucella* a viable threat to public health. One of the main impediments to vaccine development is identification of suitable antigens. In order to identify antigens that could potentially be used in a vaccine formulation, we describe a multi-step antigen selection approach. We initially used an algorithm (Vaxign) to predict ORF encoding outer membrane proteins with antigenic determinants. Differential gene expression during acute infection and published evidence for a role in virulence were used as criteria for down-selection of the candidate antigens that resulted from *in silico* prediction. This approach resulted in the identification of nine *Brucella melitensis* outer membrane proteins, 5 of which were recombinantly expressed and used for validation. Omp22 and Hia had the highest *in silico* scores for adhesin probability and also conferred invasive capacity to *E. coli* overexpressing recombinant proteins. With the exception of FlgK in the goat, all proteins reacted to pooled sera from exposed goats, mice, and humans. BtuB, Hia and FlgK stimulated a mixed Th1–Th2 response in splenocytes from immunized mice while BtuB and Hia elicited NO release from splenocytes of S19 immunized mice. The results support the applicability of the current approach to the identification of antigens with immunogenic and invasive properties. Studies to assess immunogenicity and protective efficacy of individual proteins in the mouse are currently underway.

## Introduction

Brucellosis is a zoonotic, chronic and debilitating systemic disease of wide distribution but particularly important in European countries along the Mediterranean, parts of Africa and Asia, the Middle East and Central and South America [1], [2]. Animals and their food products are the primary source of human infection where the mucosal routes are the most common modes of transmission through inhalation and ingestion of animal-derived contaminated materials, respectively [1], [2], [3], [4], [5], [6], [7]. Consistent with the food animal-human symbiotic relationship, brucellosis is one of the most common zoonotic diseases estimated at an annual incidence of 500,000 cases [8]. *Brucella*, the etiologic agent, is a Gram negative, facultative intracellular bacterium classified based upon host preference where goat, swine, and cattle are preferred by the most common agents implicated in human brucellosis *Brucella melitensis*, *Brucella suis,* and *Brucella abortus,* respectively [8], [9], [10], [11], [12]. The ability to efficiently infect through mucosal surfaces and persist in the host, its highly infectious nature and the insidious, systemic, chronic, debilitating aspects of the disease are features that make *Brucella* a viable threat to public health while earning it recognition by the CDC as a select agent with potential use in a bioterrorist act [13]. At a time of emerging and re-emerging disease and bioterrorist threats, the search for protective measures in the form of novel, safe and effective prophylactic and treatment options to mitigate effects of brucellosis is a challenge that needs to be urgently addressed.

The premise that vaccination is the single most important approach to counteract infectious disease coupled with the threat represented by *Brucella* has directed research efforts towards development of a vaccine safe for human use. Based on the comparative success of live-attenuated vaccines against intracellular pathogens, including *Brucella*, research efforts have focused on the search for safer, live-attenuated vaccine candidates while the search for immunogenic and protective subunit antigens is an area that lacks, but merits, attention [13], [14], [15], [16], [17]. Consistent with findings in *Brucella* research, one main reason for the difference in conferred protection lies in the superiority of live-attenuated organisms to stimulate effective adaptive immune responses, particularly a T-cell response [14], [17]. Deciphering the mechanisms at play could potentially guide the design of a subunit vaccine. Although attenuated, live intracellular organisms retain a residual capacity to invade where they may produce an array of potential immunogenic targets that are important for intracellular adaptation, survival and replication [14], [18], [19]. The value of using antigens that are over-expressed in the intracellular compartment has been previously demonstrated [18], [20]. In contrast, the target-less traditional approach to antigen selection where subcellular fractions of *in vitro* cultivated organisms are evaluated for protective efficacy undermines the quantitative, qualitative and functional properties of the antigen preparation [21]. The availability of genomic sequences and development of high throughput genomics-dependent analytical methods such as transcriptomics, proteomics, antigenomics and immunomics are tools that could potentially accelerate development of targeted approaches for identification of subunit antigens [22]. Accordingly, the availability of genomic sequences for several important pathogens, including *Brucella,* has made the development of genomics-based analytical tools to accelerate identification of subunit immunogens possible [21].

Reverse vaccinology is a method that has yet to be applied to *Brucella* but has proven useful in the identification of protective antigens against other important pathogens [22]. Reverse vaccinology is a concept that was first introduced in 2000 and is based on the *in silico* genome analysis targeted at the identification of antigens with desirable immunogenic, structural, or functional characteristics [21], [22]. In the current report, we use a reverse vaccinology approach to select open reading frames (ORF) encoding pre-determined immunogenic, functional, and structural properties. Expressed encoded proteins and previously reported functional genomics data were used to validate the predictive ability of the analytical system.

## Materials and Methods

### Ethics Statement

All animal experiments were performed with the approval of the Texas A&M University Institutional Animal Care and Use Committee (AUP# 2010-0167). The use of unidentified human serum samples was approved by the Institutional Review Board at Texas A&M University (IRB protocol #2012-0412) based on 45 CFR 46.101(b)(4), from the US code of federal regulations.

### Antigen Selection

Antigens were initially selected using *in silico* analysis of the *Brucella spp*. genomes via the publicly available Vaxign program (vaxign.com) [23]. The initial screening identified 27 candidate ORFs. With the aid of published reports, the candidate ORFs were further selected if they were co-regulated with known *Brucella* virulence factors or had direct evidence for a role in pathogenesis. The use of pathogenesis-associated bacterial factors as antigens in vaccine preparations have been shown to confer protective immunity against *Brucella* and other important intracellular pathogens [24], [25], [26], [27]. This resulted in 9 candidate ORFs. As further evidence for a role in *Brucella* pathogenesis, global transcriptomic data in *in vivo* and *in vitro* models of brucellosis were used to verify that candidate ORFs were differentially expressed during the acute phase of infection.

### PCR Amplification Cloning and Transformant Selection

Primers were designed for the full-length amplification of selected genes (Table 1). Open reading frames were amplified by polymerase chain reaction (PCR) from *B. melitensis* 16M genomic DNA with the PCR failsafe system (Epicentre Biotechnologies, WI, USA). PCR amplicons were cloned into pET-SUMO and pTrc-His vectors (Invitrogen, Carlsbad, CA.) using TA-TOPO cloning technology. Constructs were introduced into Mach-1 *E. coli* competent cells. After verification with restriction digestion and/or sequencing, plasmid from positive transformants was obtained and used to transform BL-21 (DE3) competent cells for protein expression.

**Table 1:** Brucella melitensis 16M Genes and Proteins.

Gene	Forward Primer	Reverse Primer	Protein	MW (Da)
BMEI0454	5′-ATGGCAGCCACCGCGCTC-3′	5′-TCAGAAACGGTAGGTAATACCGGTG-3′	OmpW	23, 541
BMEI0536	5′-ATGAACACTCGTGCTAGCAATTTTCT-3′	5′-TTACTTGATTTCAAAAACGACATTGACCGATACG-3′	Bp26	26, 552
BMEI0657	5′-ATGGCGCAGGATGGCGGGGATAAG-3′	5′-TCAGAAATTACGGGTCAGCCC-3′	BtuB	64, 807
BMEI0717	5′-ATGGGAGGGACCGACTACACCTATAAC-3′	5′-CTAGAATTTGTAGTTCAGGCCG-3′	Omp22	19, 448
BMEI1872	5′-ATGCCGATGGCCCGTCATC-3′	5′-TTAATTGAAGGTATAGCTGAAACCTGCC-3′	Hia	37, 461
BMEII0160	5′-ATGTCACTTAGTTCTGCTCTTCTGACGG-3′	5′-TCACACCGCGTTAAGAAGATCATC-3′	FlgK	50, 596

### Protein Expression

BL-21 *E. coli* cells transformed with the pET-SUMO-BME and pTrcHis-BME constructs were grown to the log phase in Luria Bertani broth (LB) supplemented with antibiotic and, when appropriate, 1 mM glucose. For protein expression, log-phase BL-21 bacteria expressing SUMO-fusion protein and his-tagged protein were induced with 1 mM IPTG and incubated for 2.5 hours or 4.0 hours at 37°C, respectively. Following expression, bacteria were pelleted and stored at −20°C until used. Protein solutions and bacterial lysates were diluted with 2× SDS-PAGE sample loading buffer and electrophoresed in 12.5% (w/v) SDS-PAGE gels. Proteins were stained with Coomassie Blue to confirm their size and level of purity.

### Inclusion Body Isolation and Solubilization

OmpW, Omp22, Metal chelate Rc (BtuB), and Cell surface protein (Hia) were primarily produced as inclusion bodies. With slight modifications, inclusion bodies were purified as previously described [28]. Briefly, bacterial pellets were suspended in native purification buffer (NPB: 500 mM NaCl, 50 mM H_2_PO_4_, pH 8.0) with lysozyme, incubated at room temperature and sonicated. The resulting lysate was centrifuged at 5,000×g and the pellets were re-suspended in cold PBS. This suspension was centrifuged at 10,000×g for 30 minutes and the recovered pellet was re-suspended in PBS containing Triton X-100. This suspension was centrifuged at 22,000×g for 15 minutes. The pellet recovered was washed 3 times with PBS containing Triton X-100 and once with PBS. The pellets were stored at −20°C until used. Prior to purification, the pellets were solubilized in guanidinium lysis buffer (6M guanidine HCl, 20 mM sodium phosphate, 500 mM NaCl, pH 7.8) at 37°C.

### Gel Filtration Chromatography and Refolding

Solubilized inclusion bodies or protein expressed in soluble form were size-separated using Sephacryl S-300 resin (GE Healthcare, WI, USA) via and eluted using 4M urea or native purification buffer, respectively. Fractions were analyzed for purity via SDS-PAGE, and the urea was subsequently removed using dialysis against 1× or 2× NPB, filter sterilized and then stored at −80°C until use. Protein concentration was determined with the Bradford-based Bio-Rad protein assay (Bio-Rad, CA, USA).

### Invasion Studies

MLE-12 (ATCC, VA, USA), alveolar epithelial type II (AEII) cells were plated at 2.5×10^5^ cells/well in 0.5 ml of DMEM media and incubated overnight at 37°C with 5% (v/v) CO_2_. The following day, cells were infected with BL-21 cells containing pTrcHis-Brucella ORF constructs with or without IPTG stimulation for the expression of *B. melitensis* recombinant outer membrane proteins. After 30 minutes of incubation, gentamicin was added at 100 µg/ml and incubated for 30 minutes to kill extracellular bacteria. Cells were washed with PBS and subsequently lysed with 1% Triton X-100 in PBS. Lysate was serially diluted and bacteria enumerated by plating portions of serial dilutions on LB agar. Invasion rate is calculated using the ratio of bacteria recovered from the cells by the number of bacteria inoculated. Results were done in triplicate and the experiment was repeated three times.

### Western Blot

Pooled serum samples from 3 goats, >5 BALB/c mice, and 2 humans previously exposed to wildtype *Brucella* were pre-absorbed with BL-21 *E. coli* lysate over-expressing the SUMO-CAT fusion protein to eliminate background and, subsequently, used as probes in Western Blot analysis. BL-21 *E. coli* lysates overexpressing *B. melitensis* 16M recombinant outer membrane SUMO-fusion proteins were electrophoresed in a SDS-PAGE gel. Separated proteins were electroblotted in a wet system to nitrocellulose membrane and blocked with 5% (w/v) non-fat dry milk in PBS-T (0.1% (v/v) Tween 20 in PBS). Pooled serum samples from BALB/c mice (1∶1000), goats (1∶500) and humans (1∶1000) were diluted in PBS-T+5% (w/v) NF milk and the membranes were incubated in this mixture overnight at 4°C and then washed 3 times with PBS-T to remove excess antibody. HRP-labeled secondary antibody specific to the primary antibody was diluted 1∶1000 and incubated with the membrane. Following extensive washing, the signal was induced by the addition of substrate (Hy-Glo chemiluminescence system) according to the manufactures instructions and captured using autoradiography film (Denville Scientific Inc, NJ, USA). Images were stored as TIFF files and band intensity quantified using the *imageJ* software (NIH, MA, USA). The use of human serum samples was approved by the Texas A&M University Institutional Review Board (IRB protocol #2012-0412).

### Endotoxin Depletion

Frozen pellets were rapidly thawed using a 37°C water bath and endotoxin depleted with endotoxin-depleting columns according to manufacturer’s instructions (Norgen Biotek Corp, Canada). Depletion of endotoxin was verified with the ToxinSensor Chromogenic LAL endotoxin assay according to the instructions of the manufacturer (GenScript, NJ, USA).

### Animals and Immunization Protocol

Mice were obtained from Jackson laboratories (ME, USA) and housed in approved facilities. Groups of 3 C57BL/6, female mice were inoculated subcutaneously with PBS, ISCOMATRIX (CSL, Parkville, Australia), 10 µg S19 lysate adjuvanted with 12 µg ISCOMATRIX, or a protein cocktail consisting of 10 µg each Hia, FlgK, and BtuB adjuvanted with 12 µg ISCOMATRIX. Animals were similarly boosted 3 weeks post-priming and euthanized three weeks following boosting for splenocyte analysis. All experiments were performed with the approval of the Texas A&M University Institutional Animal Care and Use Committee (AUP# 2010-0167).

### Primary Splenocyte Culture and Cytokine and Nitric Oxide Release

Spleens from immunized and control mice were aseptically removed and processed for single cell suspension. Splenocytes were added to 96 well plates at 1.0×10^5^ cells/well in 200 µl RPMI media. The cells were stimulated with 7 µg of corresponding protein and incubated at 37°C for 24 hours. Supernatants were collected and stored at −20°C until use. Cytokines in the supernatants were analyzed with the Bio-Rad bioplex system using a Luminex 200 analyzer according to the manufacturer’s instructions (Bio-Rad, CA, USA). Analysis of IL-17A, was performed via ELISA according to the manufacturer’s instructions (eBioscience, CA, USA). Nitric oxide release was measured using the Griess reagent system per the instructions of the manufacturer (Promega, WI, USA). Surplus splenocytes were frozen and stored in −80°C until use. When the cytokine level was below the lowest value recorded for the standard, the sample was arbitrarily assigned the lowest value of the standard range.

#### Statistics

An unpaired Student’s *t*-test was used to calculate significant difference.

## Results

### Antigen Selection Strategy

Antigens were selected using *in silico* analysis of the *Brucella spp*. genomes via the publicly available Vaxign program (vaxign.com) [23]. Options for the *in silico* screening of the Vaxign program include subcellular location, presence of T cell epitopes, adhesin probability, presence or absence of orthologs in selected bacterial strains, and similarity to host proteins. Using the *Brucella melitensis* 16M genome as the reference, selection criteria for the present study included open reading frames that coded for proteins present in the outer membrane due to the importance of the outer membrane in *Brucella* pathogenesis, contained MHCI and MHCII epitopes in mouse and humans, have adhesin potential, have orthologs present in *Brucella suis* and *Brucella abortus*, and lastly, proteins with no similarity to human, or mouse proteins. With the described settings, initial screening resulted in 27 candidate ORFs. The number of candidate antigens was reduced through the selection of only those ORFs that had been ascribed, through published reports, a direct or indirect role in virulence of *Brucella* spp. Bacterial factors that support infection of *Brucella* spp. and other intracellular agents have been demonstrated to have protective value against infection when used as immunogens as indicated above [24]. First, published transcriptomics and proteomics reports on *virB* and *BvrR-BvrS* two-component system, two important virulence factors of *Brucella spp*., were used [29], [30], [31], [32]. Open reading frames encoding proteins co-regulated with these virulence factors were included for further analyses. Direct evidence was defined as a gene or protein for which a direct role in the invasion or adaptation of *Brucella* has been defined or orthologs in other pathogens for which a role in virulence has been established. *Brucella spp*. possesses a Hia autotransporter with homology to an adhesin and colonization factor in *Haemophilus influenzae*, an important human pathogen of the respiratory tract [33]. An ABC-autotranporter, BtuB, has been observed to support survival of *Brucella melitensis* 16M in the mouse macrophage [15]. Lastly, *Brucella spp*. contains an operon that codes for a flagellum. The FlgK gene, which was predicted by the algorithm, is included in such operon. It was recently reported that this operon is required to establish a chronic infection in the mouse model of brucellosis (19, 51). Following this methodology, the resulting list consisted of 9 open reading frames.

To further support a virulence role for the antigens in *Brucella*, the dynamics of gene expression during the acute phase of infection in the HeLa cell [19] and the previously described calf ligated ileal loop model system [34], [35], [36]. Down regulation of gene expression following host cell internalization, relative to inoculum, is consistent with a role in invasion while induction of gene expression is consistent with a role in adaptation of the bacterium to the intracellular niche. From among the 27 proteins initially identified using Vaxign, nine were identified that matched the additional criteria applied and were prepared for evaluation via expression in *E. coli*.

### Protein Expression and Purification

**Figure 1:**
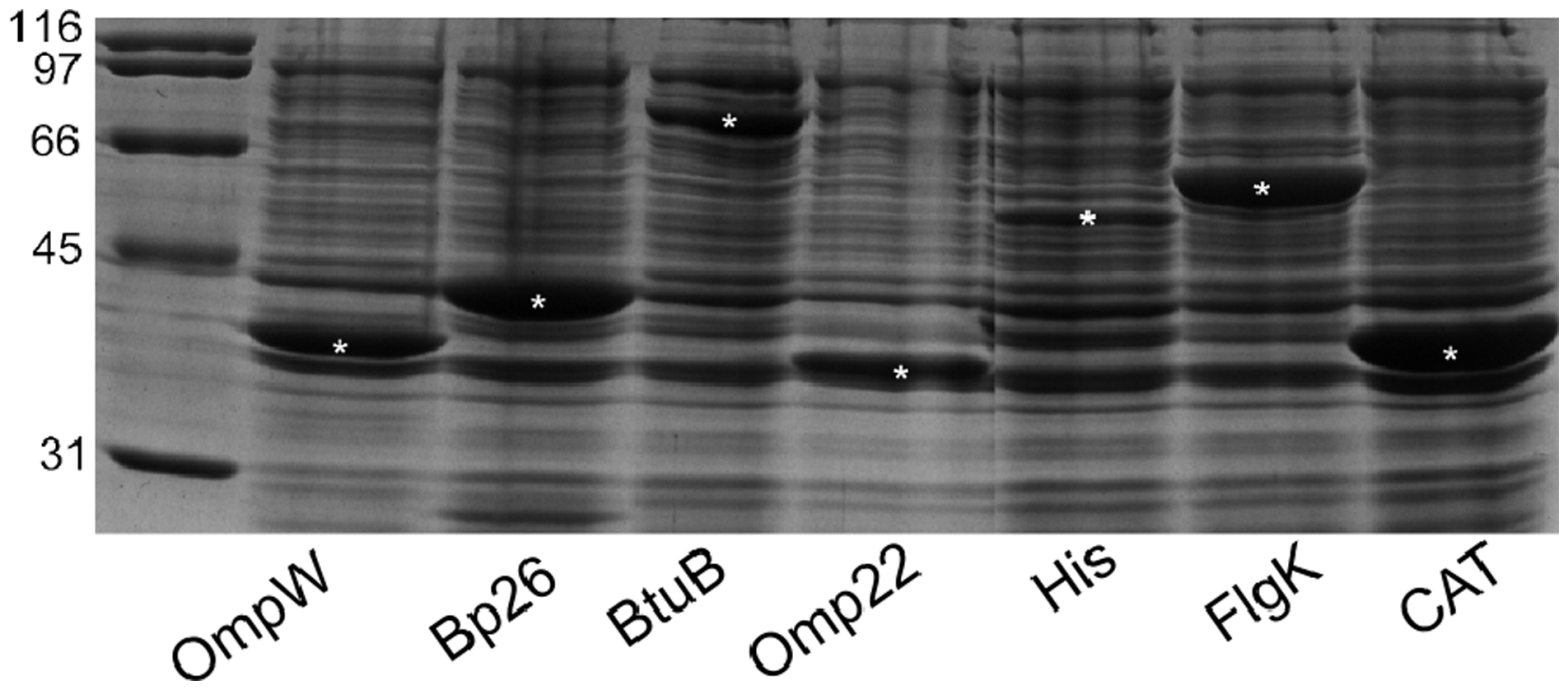
Whole cell lysate from BL-21 *E. coli* transformed with pET-SUMO-BME constructs. Bacteria were grown to log phase in LB broth supplemented with antibiotic and then stimulated with IPTG for 2.5 hours. Bacterial lysates were diluted with SDS loading buffer, incubated at 95°C for 5 minutes and electrophoresed in a 12.5% SDS-PAGE gel.

**Figure 2:**
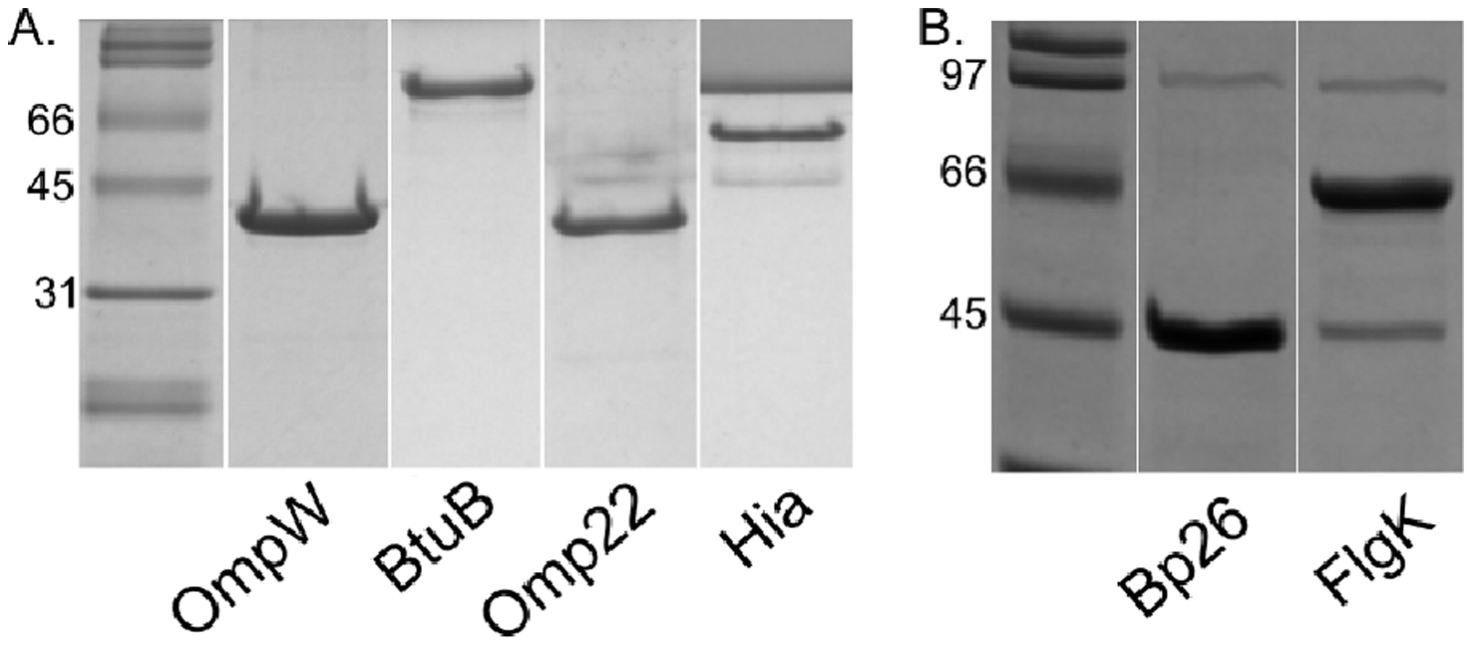
Purified *Brucella melitensis* 16M outer membrane proteins expressed as SUMO-fusion proteins in BL-21 *E. coli* cells. OmpW, BtuB, Omp22, and cell surface protein expressed in inclusion bodies were purified by centrifugation, solubilized with guanidine HCl, and separated in gel filtration column. Bacterial lysates containing BP26 and FlgK in the soluble fraction were directly purified in an S-300 gel filtration column. Purified protein fractions were diluted with SDS loading buffer, incubated at 95°C for 5 minutes and electrophoresed in a 12.5% SDS-PAGE gel.

**Figure 3:**
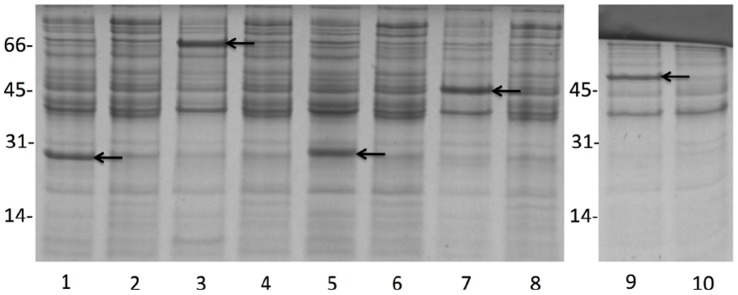
Bacterial lysates from BL-21 *E. coli* transformed with pTrc-BME constructs and used as inoculums in mouse alveolar type II epithelial cells invasion assays. Bacteria were grown to log phase in LB broth supplemented with antibiotic followed by the addition or exclusion of IPTG and 4 hours of continued incubation. Bacterial lysates were diluted with SDS loading buffer, incubated at 95°C for 5 minutes and electrophoresed in a 12.5% SDS-PAGE gel. Lanes 1, 3, 5, 7, 9, lysate from BL-21 cells stimulated with IPTG and overexpressing OmpW, BtuB, Omp22, cell surface protein, and FlgK (arrows), respectively. Lanes 2, 4, 6, 8, 10, lysates from non-stimulated BL-21 cells containing protein expression constructs for OmpW, BtuB, Omp22, cell surface protein, and FlgK.

Protein expression was induced in BL-21 *E. coli* cells harboring either pET-SUMO-BME or pTrcHis-BME constructs. Log phase bacterial cultures were treated with 1 mM IPTG to stimulate protein expression that was evaluated at several time points post-induction. OmpW, BtuB, Omp22, Hia, and Flgk were successfully expressed at sufficient levels as SUMO-fusion proteins by 2.5 hours post-stimulation (Fig. 1). Of 9 candidates selected for expression, 5 proteins were expressed at levels permitting analysis of their vaccine potential (Figs. 1 and 2). Proteins were optimally expressed under the control of the pTrc promoter by 4 to 4.5 hours post-IPTG stimulation (Fig. 3).

### Recombinant B. melitensis Omp22 and Hia Confer Invasive Properties on Non-invasive BL-21 E. Coli Strain

**Figure 4:**
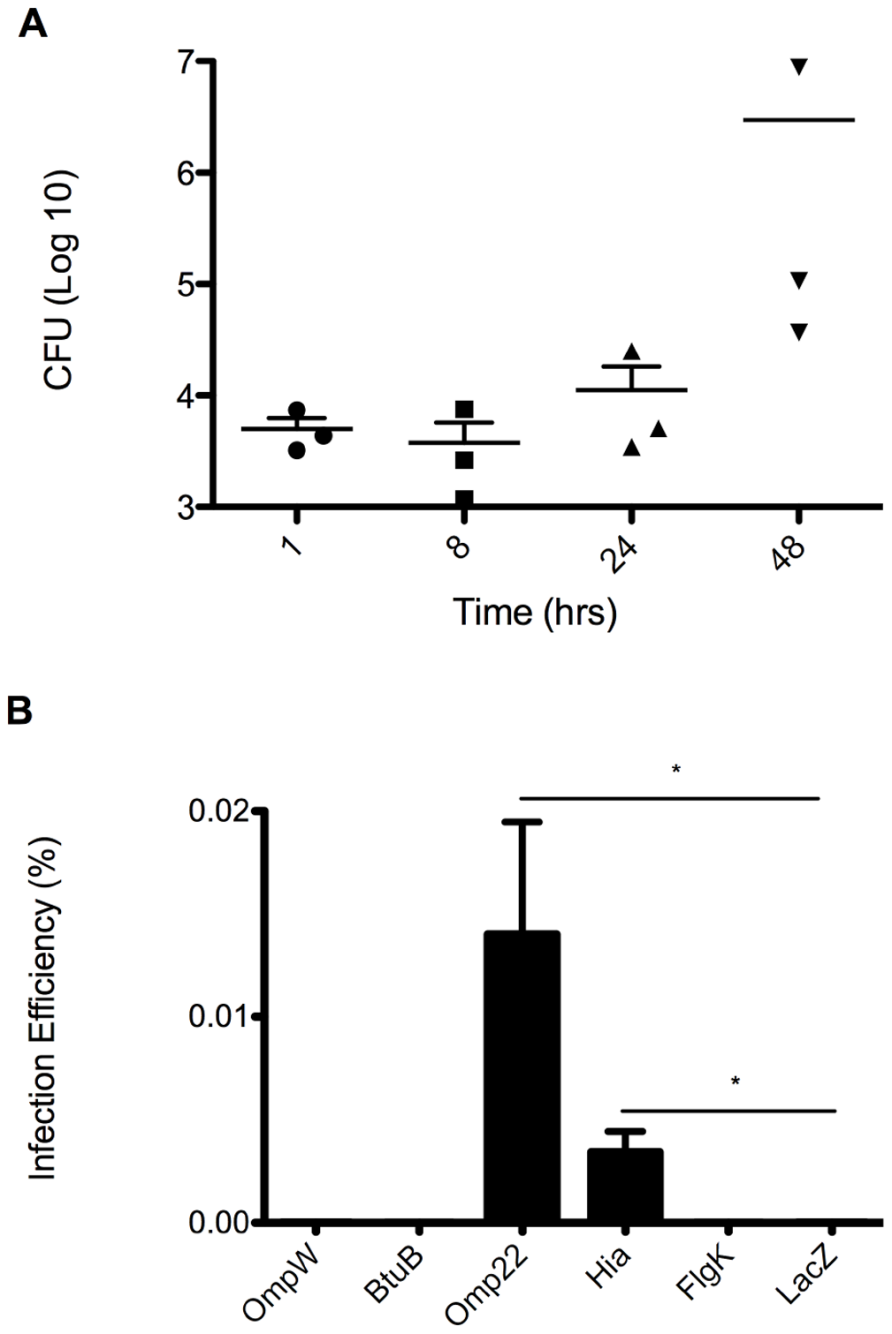
Invasion of mouse alveolar type II epithelial cells (MLE12) with *Brucella abortus* S19 (A) or BL21 E. coli overexpressing *Brucella melitensis* 16M outer membrane proteins (B). A. Mouse alveolar MLE12 epithelial cells were plated at 2.5×10^5^ with DMEM media. The following day, cells were infected with log phase *B. abortus* S19 or BL21 E. coli +/− IPTG stimulation at an MOI of 500∶1. Extracellular bacteria were eliminated in a 30-minutes incubation period in the presence of gentamicin that followed a 30-minute invasion period. Mouse cells were lysed and plated in LB with ampcillin or TSA media. *B. abortus* invasion is reported as number of bacteria recovered at different time-points. BL-21 invasion as reported as the number of bacteria recovered after 30 the minute invasion divided by the number of bacteria inoculated. Data represent the mean +/− SEM from three independent experiments in triplicate. Statistical significance was determined with one-tailed Student’s t-test (*P<0.05).G.

Based on the premise that selection of candidate outer membrane proteins included an elevated probability of identifying adhesins, a role for candidate proteins in invasion of mouse alveolar type II epithelial cells was explored. Given that the aerogenous route is a highly effective and common portal of *Brucella* infection, efforts focused on a determination of mouse alveolar epithelial cell support of infection and replication of *Brucella*. To assess this possibility, *B. abortus* S19 was used to infect the type II alveolar epithelial cell line, MLE-12 in gentamicin protection assays. MLE-12 cells infected at an MOI of 500 as described in the Materials and Methods section. The capacity to infect alveolar epithelial cells was confirmed by consistent, intracellular bacterial recovery following infection. Bacteria numbers were slightly decreased by 8 hrs post-infection but progressively increased by 24 and 48 hrs, exceeding the level of bacteria uptake (Fig. 4A) and supporting the potential contribution of these cells to infection and persistence. The results shown are inclusive of three independent experiments performed in triplicate. To determine whether the capacity to infect ATII cells was derived from any of the candidate immunogens non-invasive BL-21 *E. coli* strain over-expressing individual *Brucella* outer membrane proteins were evaluated for infection of ATII cells (Fig. 3). Following a 30-minute infection period, only BL-21 *E. coli* cells over-expressing Omp22 and Hia were able to enter epithelial cells. Compared to BL-21 cells expressing the non-relevant LacZ protein, Omp22 and Hia conferred invasive capacity on the non-invasive BL-21 cells (Fig. 4B).

### Mice, Goats and Humans Exposed to Wild Type Brucella spp. Mount Antibody Responses to Brucella melitensis 16M Outer Membrane Proteins

**Figure 5:**
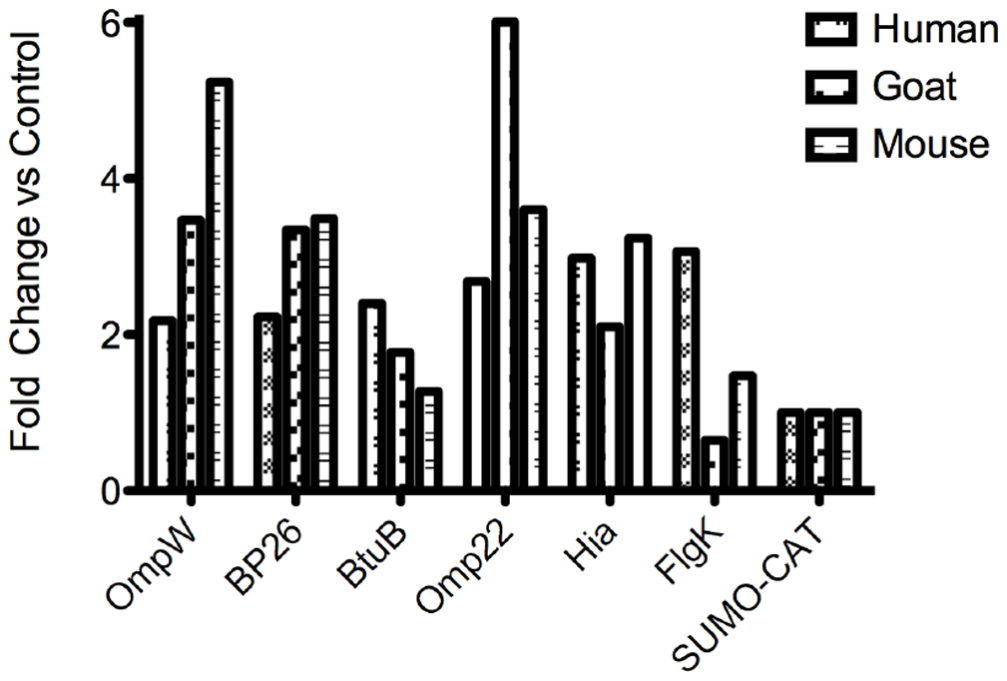
Western blot of pooled serum from exposed goats, mice, and humans to recombinant Brucella melitensis 16M outer membrane proteins. BL21 *E. coli* lysates overexpressing Brucella outer membrane proteins were electrophoresed and transferred to a nitrocellulose membrane. Recombinant proteins were probed with serum absorbed against E. coli lysates overexpressing SUMO-CAT. Secondary antibodies labeled with HRP were used and cheluminescence used for signal development. Signals were developed in autoradiography film (A) and captured as Tiff images and analyzed with *imageJ* software (B). To determine fold change, signal intensity against *Brucella* proteins bands was divided by the intensity produced against control protein SUMO-CAT.

To evaluate the humoral immune response against selected candidate antigens, pooled sera derived from wildtype *Brucella spp*.- exposed goats, BALB/c mice and humans was pre-absorbed using *E. coli* lysate and used to evaluate expression of *B. melitensis* 16M recombinant outer membrane proteins in *E. coli* lysates by Western Blots. Antibody reponses directed against the majority of the proteins were observed (Fig. 5, panels A and B). With the exception of FlgK probed with goat sera, all proteins were reactive with sera from exposed humans, goats, and mice. BP26, a known immunodominant protein, was used as positive control. While the sera from goat and mouse produced a greater signal against major Group 3 membrane proteins Omp22 and OmpW, human sera exhibited a greater response to non-Group 3 membrane proteins Hia and FlgK (Fig. 5, panels A and B).

### B. melitensis 16M Recombinant Outer Membrane Proteins Elicit Nitric Oxide Release from Splenocytes

**Figure 6:**
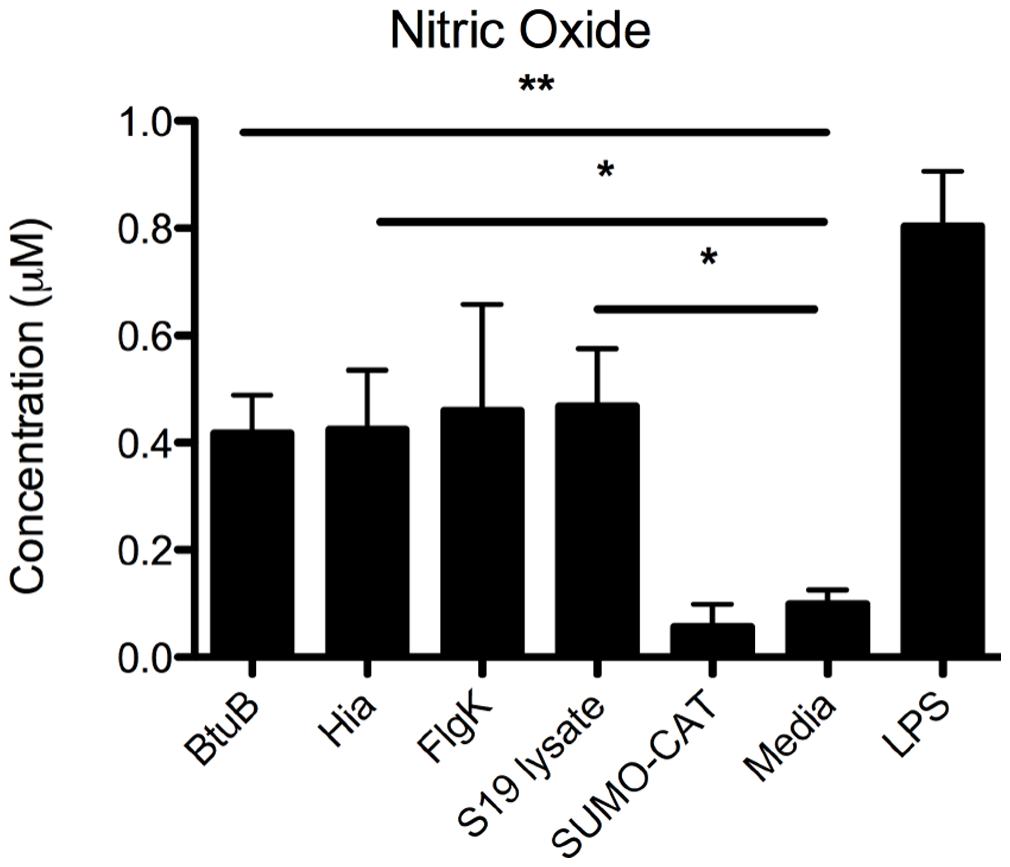
Nitric oxide release from splenocytes derived from mice inoculated with S19 lysate and stimulated with Brucella outer membrane proteins. A group of three C57BL/6 mice were inoculated subcutaneously with S19 lysate twice three weeks apart and humanely euthanized 3 weeks after the last inoculation. Spleens were harvested and processed into a single cell suspension. Splenocytes were plated and stimulated with individual *Brucella* outer membrane proteins BtuB, Hia, and FlgK. Supernatants were collected 24 hrs post-stimulation and analyzed for nitric oxide release using the promega griess reagent system. Data represent mean +/− standard error of the mean from 3 mice. Statistical significance between PBS control and treatment groups was determined with one-tailed Student’s t-test (*P<0.05, **P<0.01).

Nitric oxide (NO) plays a role in multiple physiologic functions including immunogenic responses and brucellacidal activity [37], [38], [39], [40]. To investigate whether candidate *Brucella* outer membrane proteins elicit release of nitric oxide, single recombinant outer membrane proteins of *Brucella melitensis* 16M were used to stimulate primary splenocytes derived from S19 lysate-immunized mice and NO release quantified. The *B. abortus* S19 lysate, BtuB and Hia, but not FlgK, elicited release of significant nitric oxide from S19 lysate splenocytes (Fig. 6).

### B. melitensis 16M Outer Membrane Protein-mediated T Cell Cytokine Production in Splenocytes

**Figure 7:**
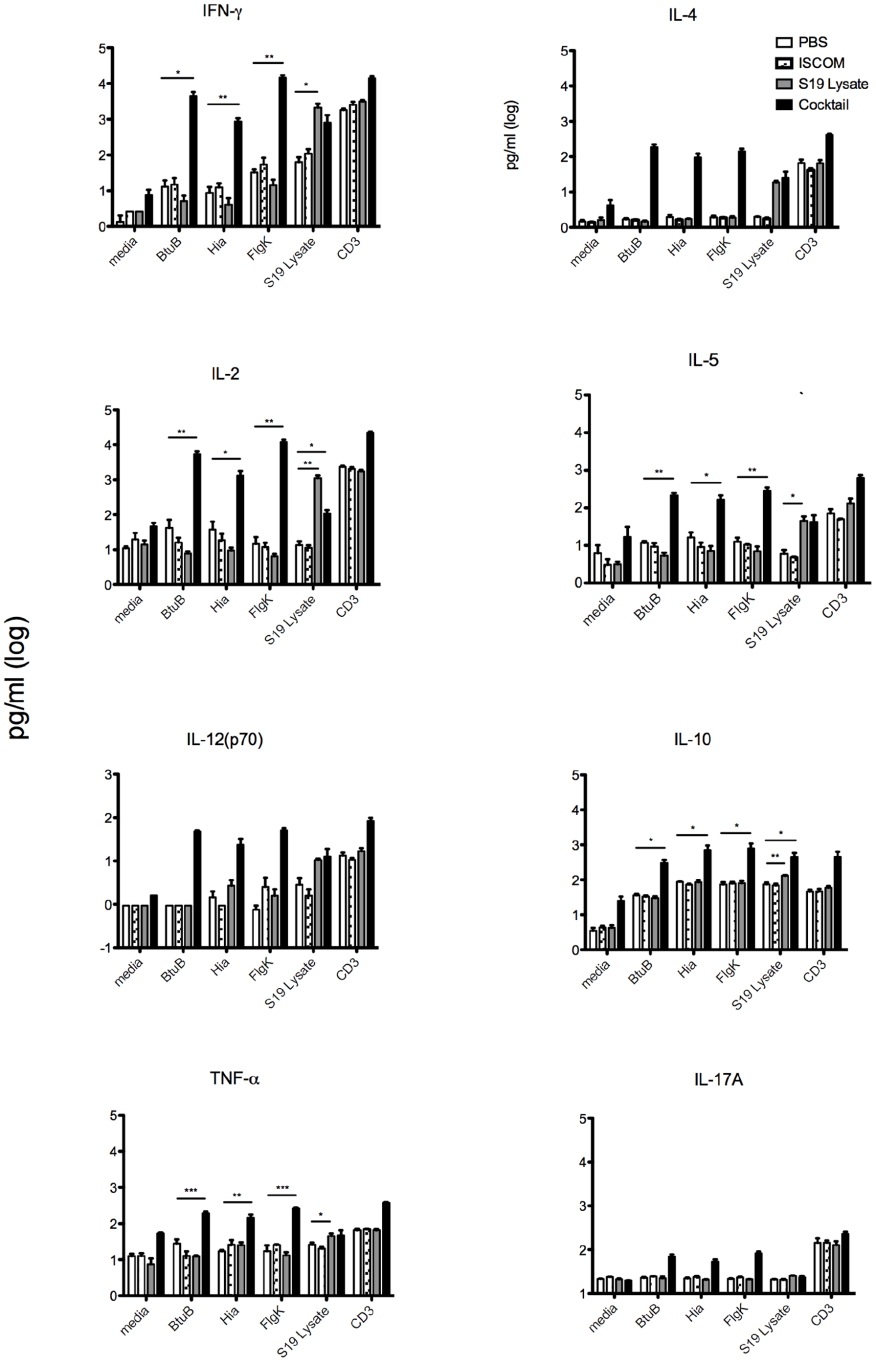
Cytokine production in splenocytes derived from mice inoculated with a cocktail of proteins BtuB, Hia, and FlgK, *B. abortus* S19 lysate, ISCOMTRIX adjuvant, or PBS. Groups of 3 C57BL/6 mice were inoculated subcutaneously twice three weeks apart and humanely euthanized 3 weeks after the last inoculation. Spleens were harvested and processed into a single cell suspension. Splenocytes were plated and stimulated with individual *Brucella* outer membrane proteins, S19 lysate, ISCOMATRIX adjuvant or RPMI media. Supernatants were collected 24 hrs post-stimulation and analyzed for cytokines using the Th1/Th2 bioplex kit or indirect ELISA (IL-17A). Data represent mean +/− standard error of the mean from each group of 3 mice. Statistical significance between PBS control and treatment groups was determined with one-tailed Student’s t-test (*P<0.05, **P<0.01, ***P<0.001).

The three highest scoring proteins with regards to predicted human and mouse MHC epitopes were selected to determine antigen specific T-cell responses (Table 2). Cytokine responses of splenocytes derived from mice immunized with a cocktail of proteins were antigen specific as those responses were determined *in vitro* after stimulation of immune splenocytes with individual antigens. Furthermore, preliminary results in our laboratory indicate that S19 lysate inoculation induces protective immunity against *Brucella* (unpublished data). Inoculation with a cocktail consisting of the highest scoring antigens in this study was an effort to emulate the heterogeneity of the S19 lysate formulation. Groups of three mice were primed and boosted three weeks apart with a cocktail of BtuB, Hia, and FlgK recombinant proteins or S19 lysate formulated with ISCOMATRIX adjuvant. Three weeks after boosting, spleens were collected and processed for primary splenocyte culture. Splenocytes were stimulated with single recombinant protein and culture supernatant collected to determine antigen-specific cytokine responses. Cytokine analysis resulting from antigen-specific stimulation was reflective of a hybrid Th1–Th2 response. Although a mixed T-cell response, the magnitude of secreted Th1 derived cytokines, IFN-γ and IL-2, was greater than that represented by a Th2 response, IL-4, IL-5 and IL-10 for all three proteins evaluated (Fig. 7). These findings support the availability of an environment conducive to a more potent Th1 than Th2 response that results from either the microenvironment created by the adjuvant-antigen complex and/or inherent immune determinants of the antigen. Primary splenocytes were also stimulated to release TNF-α in the presence of recombinant protein or S19 lysate (Fig. 7). To further characterize the immune response, IL-17 release into the supernatant was also quantified. Although to a lesser degree than for Th1 and Th2 responses, the antigen elicited significant IL-17 responses in splenocytes recovered from all recombinant proteins and S19 vaccinated mice (Fig. 7).

**Table 2:** Summary of Published and Experimental Findings.

Experiment	Gene (Protein)					
	BMEI0454(OmpW)	BMEI0657(BtuB)	BMEI071(Omp22)	BMEI1872(Hia)	BMEI0160(FlgK)	BMEI0536(Bp26)
**16M Transcriptomics**						
Bovine^a^	15 m: −2.01	15 m: +1.68	15 m: −6.95	15 m: −13.63	15 m: +2.51	N/A
	120 m: −1.72	120 m: +3.44	120 m: −8.13	120 m: −11.86	120 m: +2.69	
HeLa [19]	4 h: −9.97	4 h: +2.66	4 h: −5.63	4 h: −2.36	4 h: +2.94	N/A
	12 h: −21.03	12 h: +2.58	12 h: −6.15	12 h: −1.74	12 h: +3.7	
**16M Mutagenesis ** [15]	N/A	Attenuated	N/A	N/A	N/A	N/A
**BvrR/S- ** ***B. abortus*** **** [31], [32]	–	–	–	N/A	N/A	N/A
**Immunoreactivity** b						
Human	2.2	2.4	2.7	3	3.1	2.2
Mouse	5.2	1.3	3.6	3.2	1.5	3.5
Goat	3.5	1.8	6	2.1	0.6	3.3
**Vaxign Program ** [23]						
Adhesin Probability	0.586	0.504	0.609	0.656	0.39	N/A
Human MHCI score	0.296545	0.361865	0.263562	0.288301	0.339568	N/A
Human MHCII Score	0.32461	0.382724	0.30115	0.344729	0.337355	N/A
Mouse MHCI Score	0.341885	0.416492	0.314076	0.345491	0.392729	N/A
Mouse MHCII Score	0.310426	0.37862	0.291076	0.359367	0.353167	N/A
Outer Membrane Score	1	1	0.992	0.992	0.949	N/A
**Additional notes**				Homology toColonization	Role in Chronic	Immunogenic in
				Factor inH. Influenzae [33]	Infection in Mice [67]	Goat/Human [68]

a Rosseti, CA, et. al., manuscript in preparation.

b ImageJ analysis.

(−) gene or protein is down regulated.

16M- *Brucella melitensis* 16M.

N/A- not available.

## Discussion

Brucellosis represents a public health threat of global significance for which a vaccine capable of providing protection against human infection is lacking [41]. Consistent with the lack of vaccine, the traditional approach to the identification of subunit protective antigens is tedious and often an unrewarding endeavor [21]. Reverse vaccinology is a genomics-based *in silico* predictive tool with early success and future promise in the identification of protective antigens against important animal and human pathogens [22], [42]. Despite the surfacing of promising results, reverse vaccinology has yet to be applied to *Brucella*. In the present study, we integrated *in silico* sequence analysis, functional genomics, proteomics, and virulence-relevant data into a multi-step antigen selection approach to identify immunogenic *Brucella melitensis* 16M outer membrane protein candidates. By following this approach, we were able to identify novel candidate outer membrane proteins that conferred invasive properties to *E. coli* which suggest a role in *Brucella* invasion, elicited humoral and T cell responses, and stimulated nitric oxide release by splenocytes from mice immunized with S19 lysate.

The critical role that virulence factors play in the pathogenesis of infectious agents makes them desirable candidates for vaccine development due to their implied conserved nature [17]. Accordingly, a salient aspect of *Brucella* is its highly effective ability to penetrate host mucosal membranes and infect a variety of non-phagocytic and professional phagocytic cells [43], [44], [45], [46], [47], [48], [49]. Among susceptible cell types, the host alveolar epithelial cell is of particular interest to our laboratory due to the ability of *Brucella* to establish an infection via the aerogenous route at a relatively low dose [11]. It was previously demonstrated that the A549 human alveolar epithelial cell line is susceptible to *Brucella* infection and replication [49]. Accordingly, we demonstrated that *Brucella* also infects and replicates in mouse alveolar Type II epithelial cells, thereby, affording us a tool to evaluate invasive properties of candidate *Brucella* outer membrane proteins. In support of the predictive ability of the vaxign program, the two proteins with the highest adhesin scores, Hia and Omp22, conferred a dose dependent (IPTG induced vs. non-induced) invasive phenotype to the non-invasive BL-21 *E. coli* strain in mouse Type II cells. These results are supported by prior reports attributing an invasive function to *Brucella* Omp22 and a role in colonization to *Haemophilus influenzae* protein, Hia [33], [50]. These data suggest that *Brucella* outer membrane proteins Omp22 and Hia are successfully produced and transported to the surface of *E. coli,* as previously observed, but does not confirm proper folding of proteins or translocation of proteins lacking invasive functionality [51]. Targeted deletion of genes encoding *B. melitensis* candidate proteins is currently underway in an effort to confirm and understand their overall contribution to invasion.

Although selection of antigens associated with virulence of a pathogen is a valuable and common practice due to their implied indispensable and, hence, conserved nature, their ability to be recognized by the immune system and elicit desired responses is an aspect that must take precedence in the selection process [17]. An effective adaptive immune response is critical for protection against brucellosis. The importance of this branch of the immune system has been established by protection studies targeting the role of T-cells and humoral immunity [52], [53], [54], [55], [56], [57]. These observations are consistent with the growing evidence indicating that antibodies have a protective role against intracellular bacterial infections [58]. Despite reports of *Brucella* outer membrane proteins eliciting immune responses, characterization of their specific immunogenicity and potential contribution to protection have not been fully explored [10]. Western blot analysis indicated that serum from three *Brucella*-exposed hosts had varying IgG responses to the candidate proteins. These results indicate their ability to elicit humoral responses across host species and may be of value when targeting immunity in multiple hosts, a highly desirable feature of an immunization protocol against this important zoonotic disease.

Although evidence for a role of antibody responses in the protection against brucellosis remains a subject of controversy, an important role for T-cell responses is well accepted [59], [60]. Adaptive T-cell immunity includes activation and responses of CD4+ or CD8+ T-cells. The protection against brucellosis that is conferred by CD4+ T-cells is primarily through secretion of relevant cytokines that stimulate other immunocytes to eliminate *Brucella* [48], [56], [61]. In order to secrete cytokines, T-cells must recognize antigen in the context of MHC molecules. Th1, Th2 and Th17 are CD4+ cells that rely on the presentation via the MHCII complex [62]. Specifically, evidence exists that supports the role of Th1 and Th17 products in protection against brucellosis [48], [61], [63]. The Th1 products, IFNγ and TNF-α and the Th-1 supportive cytokine IL-12 have been correlated with protection against intracellular pathogens including *Brucella* [60], [61], [64]. On the other hand, activation of Th2 cells and secretion of associated cytokines has been linked to chronic, relapsing brucellosis [65]. Recently, a role in subunit vaccine-induced protection for IL-17 was demonstrated in mice [63]. To support *in silico* prediction and selection of antigens bearing T cell epitopes, antigen-specific recall responses from splenocytes derived from mice immunized with a cocktail of Hia, FlgK, and BtuB proteins elicited secretion of Th1, Th2 and Th17 cytokines. Although a mixed T-cell response, the premise that the magnitude of Th1 cytokine secretion exceeded that of Th2-derived cytokines justifies their inclusion in future protection studies. The adjuvant used in this study was selected based on its ability to support T-cell activation and its safety aspect. ISCOMATRIX has been shown to support Th1, Th2 and antibody responses. Additionally, the ability of ISCOMATRIX to promote cross presentation and activation of CD8+ T cells and the demonstrated role of CD8+ T cells in clearance of brucellosis also weighed on our decision to include in our studies [66]. In contrast, the widely used aluminum based adjuvants are good at promoting Th2 and antibody responses but they do not promote CD8+ T cell responses to the degree that ISCOMATRIX does [66]. One of the many roles of helper T-cell derived cytokines is to stimulate immunocytes such as macrophages to produce and secrete nitric oxide, a chemical linked to clearance of *Brucella* [37], [40]. Candidate antigen-specific stimulation resulted in significantly greater NO secretion in splenocytes derived from S19 lysate-immunized mice. Again, providing justification for their further evaluation as immunogens.

Taken together, our study indicates that *in silico* analysis provides the ability to predict *Brucella* antigens that exhibit immunogenic and invasive properties. Mouse studies including single antigen inoculations to assess immunity and protection against *B. melitensis* 16M are currently underway.
